# Clinical outcomes in transient epileptic amnesia: A 10‐year follow‐up cohort study of 47 cases

**DOI:** 10.1111/epi.17214

**Published:** 2022-03-18

**Authors:** Sharon A. Savage, John Baker, Fraser Milton, Christopher Butler, Adam Zeman

**Affiliations:** ^1^ 61554 Cognitive & Behavioural Neurology College House University of Exeter Medical School Exeter UK; ^2^ School of Psychological Sciences University of Newcastle Callaghan New South Wales Australia; ^3^ Dementia Research Centre UCL Queen Square institute of Neurology London UK; ^4^ 3286 Discipline of Psychology Washington Singer Laboratories University of Exeter Exeter UK; ^5^ 4615 Nuffield Department of Clinical Neurosciences John Radcliffe Hospital University of Oxford Oxford UK; ^6^ Department of Brain Sciences Imperial College London UK

**Keywords:** adult‐onset epilepsy, clinical outcome, cognitive impairment, comorbidities, memory

## Abstract

**Objective:**

Transient epileptic amnesia (TEA) is a form of adult‐onset epilepsy where presenting features are well described, but little is known regarding prognosis. This study aimed to elucidate the long‐term prognosis of TEA regarding seizure control, memory, medical comorbidities, and life expectancy.

**Methods:**

Up‐to‐date clinical information was collected for 47 people diagnosed with TEA who had joined the The Impairment of Memory in Epilepsy (TIME) study 10 years earlier. At entry to the study, information about comorbid conditions was systematically collected. Details regarding subsequent diagnoses, seizure activity, changes to treatment, or reports of cognitive impairment were obtained through the family doctor. The variables of interest were compared with UK population data.

**Results:**

Mortality in the cohort was 21 of 47 (45%), with an average age at death of 82.5 years. Seizures remained well controlled for the majority but medications required adjustments in dose and type for some (28%). A small number (three cases) remained seizure‐free without medication. History of cardiovascular disorders was frequent (78.7%), typically involving hypertension (55.3%). Autoimmune disorders (25.5%), cancer (23.4%), and depression (21.3%) were also commonly reported. Although persisting memory problems were often noted, dementia was diagnosed in seven cases (14.9%). Life expectancy and comorbidities in TEA did not differ from available population norms.

**Significance:**

Results suggest that life expectancy is not reduced in TEA. Although TEA does not appear to be a self‐limiting form of epilepsy, seizures are typically well controlled via medication. Because adjustments to medication may be required, even after long periods of stability, ongoing medical monitoring is recommended. Comorbid vascular disorders are frequent but appear similar to general population estimates. Monitoring mood may be important, given that people with chronic conditions are often vulnerable to depression. Because of persisting memory difficulties, the development of effective memory interventions for people with TEA is warranted.

**Key point box**

- Little research has been conducted previously regarding the long‐term outcomes in transient epileptic amnesia (TEA).
- Analysis of clinical outcomes of 47 people with TEA indicate no reduction in life expectancy.
- Seizures are generally well controlled with medication in TEA but should continue to be monitored.
- Cardiovascular disorders are the most common comorbidity with TEA, occurring in over 75% of people with TEA, but not more frequently than in the general population.
- Memory difficulties (either stable or worsening) are reported in most people with TEA, indicating a need for the development of memory interventions and support.

## 1 INTRODUCTION

Transient epileptic amnesia (TEA) is a form of adult‐onset epilepsy in which brief episodes of amnesia recur in the absence of disruption to other cognitive functions. To meet diagnostic criteria, amnesic episodes must be witnessed, and there must be supporting evidence of epilepsy via other clinical features (eg, lip‐smacking, presence of an aura), epileptiform abnormalities on electroencephalography (EEG), or through response to antiseizure medication.^1^ Using these criteria, TEA may be distinguished from other similar conditions: transient global amnesia,^2^ where typically only one, longer (eg, 4–6 h) amnesic episode occurs without evidence of epilepsy;^3^ and the more broadly defined epileptic amnesic syndrome, where pronounced interictal memory deficits may arise in the context of subtle, nonamnestic temporal lobe seizures.^4^

Following publication of the diagnostic criteria in 1998, ~250 cases of TEA have been reported.^2^, ^4^, ^5^, ^6^, ^7^, ^8^, ^9^, ^10^, ^11^, ^12^, ^13^, ^14^, ^15^ These studies have been important in identifying characteristic features, including male predominance,^15^, ^16^ onset in mid‐late adult life (on average 57–63 years), monthly frequency of attacks,^6^, ^15^ and attacks on awakening (23%^6^ to 85%^15^ of cases). Additional epileptic phenomenon, such as olfactory hallucinations,^9^ automatisms^5^ or brief spells of unresponsiveness, are commonly experienced immediately preceding or in conjunction with these amnestic attacks. Mechanisms leading to the onset of TEA appear variable, including reports of limbic encephalitis,^17^ structural abnormalities,^2^, ^5^, ^10^ or neurodegenerative disease^18^; however, in many cases the genesis remains unknown. Comorbidity with certain cardiovascular conditions (cardiac arrhythmia, cardiac valve disease, arterial aneurysm) may occur at a higher rate than in age‐matched controls.^19^ Other comorbidities reported in TEA cohort studies include autoimmune conditions and depression,^6^ although how these rates compare with population norms has yet to be established.

Treatment for TEA typically involves a low‐dose anti‐seizure medication, resulting in a high rate of seizure cessation.^6^, ^13^, ^14^, ^19^, ^20^ Although this addresses the ictal memory disturbance, many group studies comparing patients on treatment with matched healthy controls suggest ongoing cognitive sequelae in the form of patchy retrograde amnesia for salient life events,^8^, ^21^ accelerated forgetting of recently acquired memories,^22^, ^23^, ^24^, ^25^ and impairment of topographical memory.^5^ Standard neuropsychological tests of anterograde memory tend not to reveal severe impairments, with reductions either in the mild to moderate range^5^, ^26^, ^27^ or in some cases, normal performance.^1^, ^10^, ^14^, ^28^ Other neuropsychological domains tend to remain intact.^5^, ^6^

Although these characteristics of TEA are now well documented, the long‐term prognosis, with regard to seizure control, dementia risk, medical comorbidity, and seizure control has not yet been well studied. Short‐term follow‐up studies over a 6‐ to 18‐month period suggest ongoing seizure control and the potential for improvements in some aspects of memory once seizures have ceased^6^, ^9^, ^14^, ^27^ (although in some cases ongoing memory difficulty is reported despite seizure cessation).^8^, ^18^, ^25^ The patchy loss of memory for personal life events, however, appears to persist,^8^, ^9^, ^29^ with only one case to date reporting recovery of some remote episodic memories that had been inaccessible.^30^

Follow‐up information at 5 years or beyond is extremely limited, with mixed results. In one case, a man with untreated TEA experienced ongoing seizures (~12 over the 5‐year period), but maintained average performance on standard memory measures, with no evidence of general cognitive decline, and no further medical conditions reported.^29^ In contrast, a woman with a 16‐year history of untreated TEA subsequently developed Alzheimer's disease (AD).^31^ In this case, the woman initially experienced three long episodes of amnesia over the first 6 years, but then began experiencing shorter, monthly attacks for ~10 years. Once antiseizure medication was commenced, she remained seizure‐free (4 years to the end of the study), but continued to experience memory decline and met criteria for AD. The authors suggest that TEA may have been the first sign of this disease. Indeed, recent evidence from mouse models and human studies highlight the co‐occurrence of both convulsive and nonconvulsive seizures with AD, particularly in cases of familial disease.^32^, ^33^, ^34^, ^35^

In contrast, in a 20‐year follow‐up of nine cases, no indication of increased risk of dementia or other negative impact on life expectancy was found.^36^ However, neuropsychological re‐assessment after 10 years (five people)^37^ and 20 years (three people) revealed persistent memory difficulty, and in some cases, decline. With respect to epilepsy outcomes, some cases remained seizure‐free, whereas others required adjustments to medication after a period of stability (a finding reported in other cases at shorter intervals^12^). These variable accounts across a small number of people make it difficult to answer questions currently posed by the TEA community regarding whether continuous treatment is required, whether other medical conditions may be associated comorbidities, and whether further memory declines and/or dementia may be expected.

The current study provides the largest prognostic study to‐date, reporting on clinical outcomes in 47 people diagnosed with TEA, over a 10‐year period. In particular, this study aimed to provide information on:

1. Mortality and whether life expectancy with TEA differs from population norms;
2. The course of the epilepsy over the 10‐year period (eg, seizure activity and use of medication) to provide any indications as to whether this type of epilepsy is self‐limiting;
3. The prognosis with regard to dementia;
4. The prevalence of additional medical conditions with a focus on cardiovascular diseases and whether this differs from population norms.

## 2 METHOD

### 2.1 Participants

All 47 TEA participants were recruited from The Impairment of Memory in Epilepsy (TIME) database and had formed part of an initial series by Butler and colleagues.^5^ As part of entry to the original study, the diagnosis of TEA was confirmed in all cases by one neurologist (CB), with evidence of epilepsy through any combination of epileptiform abnormalities on EEG, reports of concurrent classically epileptic features (eg, olfactory hallucinations, automatisms), and/or a positive treatment response to antiseizure therapy.

The original study and follow‐up study were approved by the Multicentre Research Ethics Committee, United Kingdom (MREC 03/10/77). All participants gave written, informed consent for review of their medical record.

### 2.2 Procedure

Data regarding each participant's sex, date of birth, initial antiseizure medication (type and dose), and comorbidities at the time of the original study were extracted from the TIME database. To gain updated medical information, a request letter was sent to each participant's general practitioner (family doctor), asking for relevant consultation records for the 10‐year period following the initial study, with a particular focus on memory complaints, ongoing seizures, and other significant medical events. Copies of any correspondence with secondary care (eg, discharge summaries) or other medical specialists (eg, neurology, psychiatry/psychology, cardiology, oncology) were also requested and reviewed by experienced medically trained members of the team (AZ and JB). For cases where medical files could not be accessed due to patient death, updated information was extracted from death certificates. Patient research files were also reviewed for any additional correspondence regarding medical updates between the research team and the families over the 10‐year period. Although the initial period of follow‐up was between 2004/5 and 2015, where additional information regarding deaths became available during our analyses, these updates were included (ie, extending the period of follow‐up to 13 years for some participants).

To gain detailed information on cognitive outcomes, all participants were invited for a neuropsychological review, repeating the same battery of tests from the original study.^5^ Full results of the 14 people who were able to take part have been reported elsewhere.^37^ We summarize relevant information regarding cognitive status from this study below.

### 2.3 Data review

For each participant, a record was made of any additional diagnoses, seizure activity, or changes to medication, or reports of cognitive impairment (based upon GP reports, patient self‐report, and/or from neuropsychological review, where available). Combined with the original information, all existing and historic diagnoses for an individual were then categorized into the following major classes of medical disorder: vascular, neurological, psychiatric, malignancy/pre‐malignancy, immune‐mediated, gastrointestinal, and endocrine.

Frequencies of disorders of interest (ie, vascular, immune‐mediated, psychiatric, neurological, autoimmune, and malignancy) were calculated for the cohort and then considered in the context of UK population prevalence data obtained through: The Health Survey for England (2017),^38^ The Alzheimer's Society Dementia UK Report^39^ (the British Heart Foundation^40^), The National Institute for Health and Care Excellence^41^, ^42^), Cancer Research UK,^43^ and The Migraine Trust^44^). These sources were chosen as they provide open access, UK‐wide prevalence data regarding commonly occurring conditions that were reported in our population. As many of the conditions (including atrial fibrillation, stroke, and dementia) demonstrate an increasing prevalence with age, and age at the time of data collection varied extensively in the TIME cohort, population figures were obtained for multiple age ranges from the reference populations, where available.

To gain a rudimentary indication of whether cardiovascular conditions appeared more prevalent in patients with TEA than in the population at large, chi‐square analyses (using *p* < .05 as a threshold for significance) were conducted using available population norms. Given the limited published data regarding sample size in other clinical population cohorts, it was not possible to conduct similar analyses for other comorbid conditions. In these cases, population figures are provided for reference only.

## 3 RESULTS

Updated medical records or summary reports from GPs were obtained for 37 of the original 50 patients. In 10 additional cases, where it was not possible to locate the GP or medical record, information regarding mortality, additional diagnoses, and/or other relevant changes was obtained from the patient's family, the patient themselves, or clinical correspondence.

In the remaining three cases (two male, one female), participants were lost to contact (having moved and changed GP), and it was not possible to obtain any information regarding updated medical history or current status. As a result, these cases were removed from the analysis.

The current study is based, therefore, on 32 men and 15 women with TEA, ranging in age from 60 to 92 years (Appendix 1 for summary information on each individual case). Mean age at the time of data collection (using age at death for those deceased) was 80.2 years (standard deviation [SD] = 7.4 years).

### 3.1 Mortality

Mortality in the cohort was 21 of 47 (45%), with an average age at death of 82.5 years (SD = 6.6 years; 4 female, 17 male). UK population norms, from the Office for National Statistics,^45^ indicate that an average age at death of 81.3 years would be expected. Overall, pneumonia was the most common cause of death (*n* = 7 of the 21 patients). Other causes of death included cancer (*n* = 5; esophageal cancer, leukaemia, metastatic bronchial carcinoma, mesothelioma, Hodgkin's lymphoma), cardiovascular disorders (pulmonary embolus, ischaemic stroke, cardiac failure), end‐stage myelofibrosis, emphysematous cystitis, and AD. For six patients the cause of death was unclear from the medical information obtained.

A comparison of the characteristics and outcomes of participants within the cohort by mortality outcome showed that those who now deceased were significantly older than the remaining participants, both at the time of TEA onset (t(45) = 2.216, *p* = .03) and at the time of the original study (t(45) = 3.885, *p* < .01). There was no indication that those who were deceased were more likely to have experienced additional seizures (χ^2^ (1, *N* = 47) = 1.501; *p* = .22). As expected with increasing age and in the context of a life‐limiting condition, a higher prevalence of dementia was observed in those who were deceased (χ^2^ (1, *N* = 47) = 5.832; *p* = .02). Although there was some tendency toward a higher frequency of vascular conditions in the deceased, the difference in proportion did not reach statistical significance (χ^2^ (1, *N* = 47) = 3.189; *p* = .07) (Table 1).

**TABLE 1:** Demographic data and prevalence of health condition categories in the TIME cohort (living vs deceased)

	Total sample (*n* = 47)	Living (*n* = 26)	Deceased (*n* = 21)	*p*‐value (living vs deceased)
Age at TEA onset (years)	62.3	59.7	65.4	0.03
Age at TIME1 (years)	68.5	64.8	73.2	<0.01
Male: Female (% male)	68.1	57.6	81.0	0.10
% cases with seizures since TIME1	46.8	53.8	35.7	0.22
% cases with vascular disorders	78.7	68.0	90.4	0.07
% cases with autoimmune disorders	25.5	23.1	28.6	0.60
% cases dementia	14.9	3.8	28.6	0.02
% cases with depression	21.3	15.4	28.6	0.25

Note

TIME1 refers to initial entry to the TIME project.

### 3.2 Seizures and associated treatment

Information regarding medication use and any changes to dose or type was available in 36 participants. Approximately half (19/36) had remained on the same medication and dosage since the original study and had not experienced any further TEA episodes while on treatment (in one case, a brief period off treatment had resulted in two to three episodes, which ceased once medication was recommenced; another case had ceased medication for 8 years and then recommenced after seizures returned). An additional four people had made changes in their medication (either regarding dose or type of antiseizure), as a result of side effects (eg, tremor or fatigue) or concerns regarding interactions with other medications, but they had remained seizure‐free on treatment. A further three had tapered off and then ceased taking any antiseizure treatments and reported no further TEA attacks.

In contrast, 10 of the 36 (28%; 6 male, 4 female), had experienced some re‐emergence of seizures (in some cases these involved other seizure types such as focal impaired awareness seizure seizure or tonic‐clonic seizures) while on treatment. In six cases, these attacks emerged after a long period of stability, but resolved with an increase or change in medication type, whereas in three cases, multiple changes to the treatment were required (for one participant such changes were still ongoing at the time of interview). In the final case, the patient had chosen to cease medication 3 years earlier and reported subsequently experiencing approximately two to three episodes per year.

Monotherapy was the most common approach to treatment, with only two people initially prescribed two antiseizure medications; at follow‐up this increased to three people, one of whom was now on three medications (but had temporal lobe epilepsy preceding the onset of TEA). The drugs used as monotherapy at follow‐up (or based on last available data) were carbamazepine (*n* = 12), lamotrigine (*n* = 9), sodium valproate (*n* = 6), levetiracetam (*n* = 6), and phenytoin (*n* = 3). Combinations used in polypharmacy were sodium valproate and lamotrigine (*n* = 2) and carbamazepine, phenytoin, and levetiracetam (*n* = 1).

### 3.3 Medical comorbidities

Additional diagnoses were collated, with frequencies of each major medical condition class summarized in Table 2 and reference to population norms provided in Table 3.

**TABLE 2:** Medical conditions report in TIME cohort

	Freq
Cardiovascular	37 (78.7%)
Hypertension	26 (55.3%)
Ischemic heart disease	10 (21.3%)
Atrial fibrillation	4 (8.5%)
Stroke	4 (8.5%)
Aortic valve replacement	3 (6.4%)
Hypotension	2 (4.3%)
Transient ischemic attack	2 (4.3%)
Aortic stenosis	1 (2.1%)
Aortic aneurysm repair	1 (2.1%)
Aortic regurgitation	1 (2.1%)
psychiatric	14 (29.8%)
Dementia	7 (14.9%)
Depression	10 (21.3%)
Anxiety	1 (2.1%)
Neurological	7 (14.9%)
Migraine	4 (8.5%)
Parkinson's disease	1 (2.1%)
Peripheral neuropathy	1 (2.1%)
Gastrointestinal	4 (8.9%)
Gallstones	1 (2.1%)
Pancreatitis	1 (2.1%)
Primary sclerosing cholangitis	1 (2.1%)
Diverticulitis	2 (4.3%)
Respiratory	5 (10.6%)
Asthma	3 (6.4%)
Chronic obstructive pulmonary disease	1 (2.1%)
Obstructive sleep apnea	1 (2.1%)
Endocrine	3 (6.4%)
Type 2 diabetes	3 (6.4%)
Immune‐mediated	12 (25.5%)
Hypothyroid	4 (8.5%)
Pernicious anemia/B12 deficiency*	4 (8.5%)
Monoclonal Gammopathy	2 (4.3%)
Multinodular goiter	2 (4.3%)
Psoriatic arthropathy	2 (4.3%)
Ankylosing spondylitis	1 (2.1%)
Polymyalgia rheumatica	1 (2.1%)
Rheumatoid arthritis	1 (2.1%)
Temporal arteritis	1 (2.1%)
Malignancy/pre‐malignancy	15 (31.9%)
Prostate cancer	3 (6.4%)
Lung cancer	3 (6.4%)
Bladder cancer	2 (4.3%)
Hodgkin's lymphoma	2 (4.3%)
Breast cancer	1 (2.1%)
Colon cancer	1 (2.1%)
Leukemia	1 (2.1%)
Meningioma	1 (2.1%)
Mesothelioma	1 (2.1%)
Myelofibrosis	1 (2.1%)
Esophageal cancer	1 (2.1%)
Thyroid cancer	1 (2.1%)
Other	9 (19.1%)
Benign prostatic hypertrophy	4 (8.5%)
Glaucoma	3 (6.4%)
Chronic rhinitis	1 (2.1%)
Meniere's disease	1 (2.1%)
Osteopenia	1 (2.1%)

Note

NB: The total number of patients within each condition category may be less than the sum of all counts within categories given the comorbidities within a category).

* The cause of vitamin B12 deficiency was not reported in the available medical records. As a result, it is not known whether all cases of B12 deficiency in the cohort were related to an underlying autoimmune disease. As pernicious anemia remains the most common cause of severe vitamin B12 deficiency, a decision was made to list all cases in the autoimmune category

**TABLE 3:** Comparison of prevalence of common conditions in TEA cohort vs UK‐wide values

Condition	TEA (%)*		Health Survey for England (%)	
	<75	75+	65–74	75+
IHD	16.7	22.9	11.0 (*p* = 0.53)	16.0 (*p* = 0.28)
Stroke	8.3	8.6	5.0 (*p* = 0.60)	9.0 (*p* = 0.94)
Hypertension	58.3	54.3	58.7 (*p* = 0.98)	61.6 (*p* = 0.41)

		UK general practice 2000–2016 (%)		
		65–74	75–84	85+
AF	8.5	6	14	22

		Dementia UK report (%)				
		65–69	70–74	75–79	80–84	85–89
Dementia	14.9	1.7	3	6	11.1	18.3

		Office for National Statistics (%)
		lifetime prevalence
Depression	21.3	19.7

* Overall age range of TEA participants is 60–92 years, where *n* = 1 “55–64”; *n* = 11 “65–74”; *n* = 35 “75+”, with mean age of 80. IHD, ischemic heart disease; AF, atrial fibrillation.^38^, ^39^, ^47^, ^53^

The most common comorbidity with TEA was cardiovascular. Thirty‐seven of the 47 patients with TEA (79.%; 81% of men, 73% of women) had a documented history. Hypertension was the most commonly recorded condition, reported in 55% of patients (26/47; 6 women). Ischemic heart disease (myocardial infarction or angina) was noted in 10 of the 47 records (10/47, 21%). To facilitate comparison with data available through the NHS Health Survey for England, we divided our cohort into those younger than 75 years of age at the time of data collection (*n* = 12), and those 75 of years of age and over (*n* = 35) (Table 3). Although a numerically higher proportion of people with TEA reported comorbid ischemic heart disease (IHD) and a lower proportion of people with TEA reported comorbid hypertension, statistical comparisons with population figures indicated no significant difference (younger than 75 years IHD: χ^2^ (1, 1251) = 0.392; *p* = .53; 75 years of age and over IHD: χ^2^ (1, 1026) = 1.182; *p* = .28; younger than 75 years of age hypertension: χ^2^ (1, 885) = 0.001; *p* = .98; 75 years of age and over hypertension: χ^2^ (1, 531) = 0.719; *p* = .41).

Dementia was documented in 15% of the TEA patients (7/47, all men), a rate falling between the population estimates reported in individuals 80–84 years of age and 85–89 years of age, as seen in Table 3. In our cohort, age at dementia diagnosis was documented in five of seven cases (ages 72, 75, 76, 86, and 87; mean age of 79 years). The clinical phenotype of the dementia was noted to be consistent with AD in four patients, vascular dementia in two patients, and was unspecified in the remaining patient. Self‐reported decline in memory performance for which no formal diagnosis was given was found in a further nine participants (four women, five men). Seven of these participants were formally reassessed by the TIME project,^37^ with four found to have some objective evidence of decline on testing.^1^ In an additional four people, ongoing, stable difficulties with autobiographical memory and accelerated long‐term forgetting, or “short‐term memory” were recorded, based on self‐report or family report (with one case able to be confirmed via the completion of neuropsychological assessment).

Formal diagnoses of current or past major depression were noted in 21% of the cohort (10/47, 21%; three women and seven men), with two formal diagnoses of anxiety (2/47, 4%; one woman and one man). A further four participants had a history of some depressive or anxious symptoms within their record. Of those formally diagnosed with depression, six experienced depression prior to the onset of TEA, with two experiencing dual onset of TEA and depression. For the remaining four people, anxiety or depression arose after the onset of seizures. In reference to population estimates of lifetime prevalence, the rates of depression in both men and women with TEA appeared broadly similar to that of the general population (Women: TEA = 20% vs population = 23%; Men: TEA = 22% vs population = 17%).^46^

Autoimmune or immune‐mediated conditions were reported in 12 patients (26%). Hypothyroidism was the most common (4/47, 9%), similar to UK population rates reported in recent National Institute for Health and Care Excellence (NICE) guidance, where the prevalence of hypothyroidism in the population over 60 years of age is reported as greater than 5%.^35^ Vitamin B12 deficiency was also reported in four patients (9%), although it was not clear in all cases whether the cause of this was an underlying immune disorder. This was below the estimated prevalence of vitamin B12 deficiency (all causes) in the UK of around 20% in people older than 60 years of age.^41^

Few additional neurological conditions were reported to co‐occur within our TEA cohort. A history of migraine was reported in 9% of patients (4/47). This compares with an estimate of the global prevalence of migraine provided by The Migraine Trust of 14.7%.^37^ A range of different types of malignancy were seen (15/47, 32%; 5 women and 10 men), as compared with the Cancer Research UK report of lifetime risk of cancer of 49.7% in males, and 45.5% in females. Most commonly reported in TEA was prostate cancer (3/32 male participants, 9.4%), which was below population figures for lifetime risk of prostate cancer in males of 17.9%.^43^ No other neurological condition was reported in more than one person.

## 4 DISCUSSION

The current study is the first to report long‐term follow‐up data in a large series of patients with TEA, with information regarding broad clinical outcomes (mortality, comorbidities, including the risk of dementia) and how this form of epilepsy behaves over a 10‐year period (regarding seizures and medication).

Consistent with our smaller study in nine cases,^36^ the current results confirm within a larger group that life expectancy does not appear to be reduced, as compared with current population averages within the UK provided by the Office for National Statistics.^45^ Differences between those living and deceased within the TEA cohort appeared largely age related (as those who are deceased were on average 9 years older when they entered the TIME project). There was no suggestion that ongoing seizure activity was more likely in those who are deceased.

Reports of seizure incidence and use of medication indicated that good seizure control was experienced by the majority when on treatment. In instances where patients ceased to use medication, half experienced a re‐emergence of seizures. In addition, 10 participants experienced further seizures requiring increased doses, or changes in medication. This suggests that TEA is not a self‐limiting form of epilepsy. However, some patients experienced long periods of freedom from seizures when off medication before the attacks re‐emerged. The requirement for adjustment to medication in over a quarter of cases, even after long periods of stability, highlights the importance of ongoing medical monitoring of the condition.

As with the case study reported by Cretin and colleagues,^31^ some individuals developed a type of dementia in the years following the diagnosis of TEA. The proportion (14.9%) of cases of dementia among patients with TEA was similar to current prevalence rates for people older than 80 years of age,^39^ suggesting that there is not a strong association between dementia and TEA. Recent animal models suggest a relationship between amyloid beta accumulation and cortical excitability,^32^ but our results indicate that this is not likely to be the underlying pathology in the majority of cases of TEA. Future studies examining the presence of amyloid (eg, through the use of Pittsburgh compound B positron emission tomography scans in vivo, or analysis at autopsy) or genetic markers of dementia such as apolipoprotein E (*APOE*) status in people with TEA may shed light on the inter‐relationships between these two clinical conditions.

Persisting memory problems or declines in some forms of memory insufficient to attract a dementia diagnosis, were a common occurrence within the cohort. This is consistent with the well‐documented, high prevalence of memory difficulties reported at diagnosis of TEA.^15^ Although it is encouraging that the proportion diagnosed with dementia is not increased in comparison to the general population, and that previous work has shown stable cognitive function over 20 years of follow‐up,^36^ given that the majority continue to experience memory problems, there is a clear need to focus future research on establishing effective memory interventions for people with TEA.

Comorbid vascular disorders were frequent in people with TEA, but again the prevalence was in keeping with population statistics, available through the NHS Health Survey for England (2017).^38^ Likewise, the prevalence of autoimmune conditions (hypothyroidism and vitamin B12 deficiency), malignancy, and migraine also appeared comparable to nationwide data, although the available details of open access data for these variables prevented more detailed comparison. No clear associations between TEA and other major medical conditions emerge from this study.

A lifetime history of mood disorders was documented in approximately one fifth of the cohort. This was most commonly in the form of major depression, which for half of the cases occurred prior to TEA onset. In some instances (two cases), the onset of depression occurred at the same time as the onset of TEA. Although not qualifying for a formal diagnosis, heightened anxiety was also noted to be a feature during seizures in three participants. Although our figures are similar to the reported UK‐wide lifetime prevalence of depression of 19.7% in adults,^47^ population estimates for depression can vary widely, with an increased risk in people with chronic health conditions (such as diabetes and rheumatoid arthritis).^46^, ^48^ Moreover, several studies have described an increased prevalence of depression in people with epilepsy.^49^, ^50^, ^51^ In their systematic review and meta‐analysis, Fiest et al. report the prevalence of depression in people with epilepsy as 24.13%.^52^ One study of people with TEA reported a lifetime history of depression in 33% of their cohort, suggesting that depression frequently occurs.^6^ Although the number of cases in our sample is too small to reliably compare with population rates, we do note that people with TEA often report higher levels of depressive symptoms, using self‐report measures such as the Hospital Anxiety and Depression Scale.^5^, ^6^ This suggests that people with TEA, like others with chronic health conditions, may be more vulnerable to symptoms of psychological distress.

The overall sample size of 47 remains modest when compared with outcome studies in other conditions; however, these results provide an important step forward in knowledge regarding TEA over the long term. The current study is limited by the use of retrospective review of records, as missing information is always a possibility. Indeed, despite attempts to contact previous GPs when case information was missing or incomplete, we were unable to obtain any details for three of the original 50 participants. For a further 10 participants, information was obtained instead from the family and other clinical correspondence. For the majority of cases, however, comprehensive medical records were available, reducing any impact of this on our findings. Finally, our ability to compare the frequencies of comorbidities with established prevalence data was limited. Because our sample is small, it was not possible to conduct detailed subgroup analyses stratified by age at onset and sex.

Overall, the results of the current study suggest that TEA does not appear to limit life expectancy or increase the risk of dementia. However, regular review is advisable to ensure that medication continues to be effective, and to help manage any persisting memory or mood difficulties.

## CONFLICT OF INTEREST

The authors declare that they have no financial or other conflicts of interest in relation to this research and its publication.

## ENDNOTE

^1^
Evidence of decline was determined through the calculation of z‐scores, comparing an individual patient's test scores with a healthy control group. Negative z‐scores of −1.5 or more were interpreted as an impairment, with decline indicated by a larger negative value over time, or the appearance of a −1.5 or greater z‐score that had not previously been observed on assessment.

## APPENDIX 1

### Individual clinical outcomes for seizure history, medication, cognition, and medical comorbidities

**Table:**

ID	Sex	Living	Age/ age at death	Seizures after 2004/2005	Treatment history	Dementia diagnosis or cognitive complaint	Mood disorders	Autoimmune	Vascular
3	M	N	80	Unknown	SVP; follow‐up not available	Senile dementia (2004)	None reported	None reported	None reported
8	F	N	75	N	CBZ; ceased in 2008	None reported	Previous history of depression; self‐reported “tendency to depression”	Hypothyroid	Hypertension
9	M	Y	80	Approx 6, ongoing	CBZ, with adjusted dosages due to drowsiness (2008, 2010); change to LEV (2015)	Self‐reported memory decline (2011); moderate global atrophy on MRI brain (2014); declines on neuropsychological testing (2015)*	Depression (well before TEA onset)	MGUS	Hypertension
10	F	Y	85	Unknown	CBZ, follow‐up not available	Neuropsychological results (2015)* mostly stable performance, some reductions in immediate and recognition memory	None reported by patient	None reported by patient	None reported by patient
11	F	N	72	1 generalised	SVP, increased dosages (2005); change to LEV (2011)	None reported	Treated depression; health anxiety (subsequent to TEA onset and dx)	None reported	Hypertension, high cholesterol
12	F	Y	81	N	LAM (stable dose)	Self‐reported difficulty with route finding (2013), ongoing difficulties with ABM; performed well on neuropsychological review (2015)*	Occasional panic attack (2014); borderline anxiety on HADS (2015)	None reported	Hypertension
13	M	N	91	N	CBZ, follow‐up not available	None reported	None reported	None reported	Atrial fibrillation, hypertension
16	F	Y	92	Several	SVP+LAM; LAM dose increased due to seizures (2005)	None reported	None reported	Hypothyroid	Ischaemic heart disease, DM type II
21	M	N	76	Generalised +FIAS (at least 4)	SVP; increased dose due to seizures (2009)	Self‐report of ALF, reported largely stable and some improvement post SVP	Major depressive episode 1991 at onset of attacks	Sclerosing cholangitis, ankylosing spondylitis	Hypertension
22	M	N	71	Unknown	CBZ; follow‐up not available	Unknown	Unknown	Unknown	CVA
25	M	N	90	N	PHE (stable dose)	concerns reported by wife (2011); diagnosed Alzheimer's disease	None reported	None reported	Stroke, angina, aortic valve replacement
28	M	Y	72	3	LAM with increased doses (2006; 2009); reduced dose and ceased (2014)	Generally stable; some declines in memory observed on neuropsychological testing (2015)*	None reported	Hypothyroid	Hypertension,
31	F	Y	76	Y	SVP (stable dose)	Self‐reported difficulty with ABM, route finding; reductions in memory on neuropsychological testing (2015)*	Anxiety and depression (2001 onwards) subsequent to TEA onset and dx	None reported	Aortic stenosis
32	M	N	86	N	SVP (stable dose)	Neuropsychological assessment (2015)* showed stable cognition	None reported	Temporal arteritis	Pacemaker, hypertension
35	M	N	88	> 1	CBZ with increased dose due to seizures (2008)	No concerns reported	No history reported	B12 deficiency	Hypertension
36	M	N	91	N	PHE (stable dose)	Neuropsychological assessment (2015)* some declines and slow; confusion just prior to death	None reported	None reported	Hypertension, AF
38	F	Y	60	N	LAM (stable dose)	None reported	None reported	Family history of ankylosing spondylitis	Hypertension
39	F	Y	69	N	CBZ (stable dose)	None reported	None reported	None reported	Aortic valve replacement, CABG, IHD, hypertension, aortic stenosis
40	M	Y	83	Several	SVP with increased dose due to attacks (2013); changed to LEV due to drowsiness and tremor (2015)	memory concerns 2006; MMSE 22/30 (2014); significant memory impairments on neuropsychological (2015)*	Elevated depressive symptoms on HADS subsequent to TEA onset and dx	Hypothyroid	Hypertension
41	F	Y	80	N	LAM (stable dose)	Self‐reported “slight decline”, with ratings of little difficulty across memory tasks (2015)	None reported	None reported	Hypertension
47	M	N	80	Unknown	SVP+LAM, follow‐up information not available	None reported	None reported	None reported	Aortic stenosis
49	F	N	84	N	CBZ (stable dose), ceased in 2011	None reported	None reported	B12 deficiency	None reported
50	M	N	90	> 9	CBZ with increased dose due to seizures (2005)	Short term memory very poor (2007), diagnosis of Alzheimer's disease	None reported	None reported	Hypertension, stroke
51	M	N	74	N	SVP (stable dose); ceased 2010	Memory concerns (2007), worsening and commenced Aricept with improvement (2008), diagnosed with presenile dementia (2008);?vascular dementia	Depression (SSRI) subsequent to TEA onset and dx	None reported	Hypertension, DM, vascular event eye, angina, IHD
55	M	Y	75	N	CBZ (stable dose)	No major concerns; generally good performance on neuropsychological testing (2015)*	Depressive episode 1997 with marriage breakup, prior to TEA onset	None reported	Hypertension
56	F	Y	70	2 generalised; many “minor attacks”	CBZ +PHE; added LEV due to seizures (2008) with adjustments in dosage (2009; 2013; 2014; 2015)	None reported	None reported	B12 deficiency	Hypertension
61	M	Y	86	N	SVP (dose unchanged)	GP noted: “fairly sharp” mentally (2012); brain scan (2011,2012) said to show some atrophy; repeat (in 2014) shows ‘significant atrophy’	None reported	Rheumatoid arthritis, MGUS	TIA
62	M	Y	72	> 3	CBZ; changed to topiramate (2007); changed to LAM (2009)	Self‐reported ongoing difficulties with remote ABM	Several past episodes of major depression prior to TEA onset, some generalised anxiety; depressive episode on topiramate	None reported	None reported
64	M	Y	75	N	LAM (stable dose)	Ongoing difficulties but no reports of worsening	None reported	Psoriatic arthropathy	None reported
68	M	N	82	N	LEV (stable dose)	Worsening memory (2007); wife concerned about memory (2009); diagnosed Alzheimer's disease (2010)	some depressive symptoms (2007), subsequent to TEA onset and dx	None reported	Stroke, hypertension
69	M	Y	73	>1	CBZ; follow‐up not available	Alzheimer's disease	None reported	Unknown	Unknown
73	M	Y	86	Unknown	nil	Did not report subjective concerns	No history at original assessment	No history at original assessment	Angina, hypertension
82	M	Y	81	> 4	LAM (stable dose)	Self‐reported decline in memory but neuropsychological test results (2015)* generally stable	Borderline anxiety on HADS	None reported	Pacemaker; small vessel ischaemia
83	M	Y	88	N	LEV (stable dose)	Short‐term memory problems at time of TEA	Anxiety (treated with valium)	NONE reported	None reported
88	M	Y	74	N	CBZ; reduced dose and then ceased (2005)	None reported; ongoing difficulties with ABM	None reported	None reported	None reported
90	M	Y	87	N	LAM (stable dose)	Self‐reported ALF as worse; but neuropsychological test results (2015)* generally stable	No formal diagnosis, self‐described “depressive type”	4	Hypertension, PVD
91	M	N	89	Unknown	Nil; follow‐up not available	Poor memory noted (2012) but dementia screen revealed ‘no major concern about cognition’ (2013)	None reported	B12 deficiency	Aortic valve replacement), ischaemic heart disease, AF
92	M	N	84	Unknown	PHE (stable dose)	None reported	Depression prior to TEA onset	None reported	MI, aortic aneurysm repair, hypertension, ex‐smoker
93	M	N	82	Unknown	SVP; follow‐up information not available	Vascular dementia	None reported	None reported	AF, stroke, MI, TIA
94	M	N	83	N	CBZ (stable dose)	None reported	depression (1995) treated with SSRI at time of TEA onset	None reported	IHD, hypertension
95	F	Y	90	? 4 Unconfirmed episodes of wooziness	CBZ; dose reduced (2009)	Self‐reported concerns regarding recent memory (2015); showed poor encoding on neuropsychological testing (2015)*	None reported	None reported	Hypertension, heart attack
103	M	Y	87	N	CBZ (stable dose)	AMT 10/10 (2015) excluding dementia; no subjective concerns	None reported	None reported	Hypertension, TIA, carotid artery stenosis
112	M	Y	68	1	LAM (stable dose)	No concerns; good performance on neuropsychological tests (2015)*	None reported	None reported	None reported
114	F	Y	80	2–3 per year since ceasing LAM	LAM; ceased (2012)	None reported; good performance on neuropsychological tests (2015)*	None reported	None reported	None reported
116	M	N	74	Period of daily absences +FIAS	SVP; dose increase (2007) and addition of LEV due to seizures	2007: well above dementia cut‐off on cognitive screening (ACE‐R = 94) but ongoing ALF and ABM; reduced performance on tests of immediate recall; repeat testing 2009 no major deterioration	None reported (but anxiety noted to be a feature of attacks)	None reported	None reported
124	F	Y	77	3	CBZ; changed to LAM due to side effect (2005)	None reported (MRI in 2005 reported some frontotemporal atrophy)	None reported	None reported	None reported
138	F	N	79	Unknown	CBZ	Unknown	Unknown	Psoriasis, psoriatic arthropathy	Hypertension hypertrophic obstructive cardiomyopathy

ACE‐R, Addenbrooke's Cognitive Examination Revised; ABM, autobiographical memory; AF, atrial fibrillation; ALF, accelerated long‐term forgetting; AMT, Abbreviated Mental Test; CBZ, carbamazepine; FIAS, focal impaired awareness seizures; DM, diabetes mellitus; dx, diagnosis; HADS, Hospital Anxiety Depression Scale; LAM, lamotrigine; LEV, Levetiracetam; MGUS, Monoclonal gammopathy of undetermined significance; MI, myocardial infarction; MRI, magnetic resonance imaging; MMSE, Mini‐mental status examination; PHE, phenytoin; PVD, Peripheral vascular disease; SSRI, selective serotonin reuptake inhibitor; SVP, sodium valproate; TIA, transient ischaemic attack.

*Repeat neuropsychological assessment conducted as part of the TIME study.^37^
